# Predictors and Treatments of Proglide-Related Complications in Percutaneous Endovascular Aortic Repair

**DOI:** 10.1371/journal.pone.0123739

**Published:** 2015-04-22

**Authors:** Guohua Hu, Bin Chen, Weiguo Fu, Xin Xu, Daqiao Guo, Junhao Jiang, Jue Yang, Yuqi Wang

**Affiliations:** Department of Vascular Surgery, Zhongshan Hospital, Fudan University, Shanghai, China; S.G.Battista Hospital, ITALY

## Abstract

**Purpose:**

To investigate the predictors and treatment of the 6-Fr Perclose Proglide-related complications (PRC) in percutaneous endovascular aortic repair (pEVAR).

**Methods:**

We retrospectively analyzed the PRC after pEVAR for the treatment of aortic aneurysm or dissection in our center from December 2012 to November 2013. Procedure success was defined as effective functioning of the two devices and local hemostasis. Access-related adverse events included vascular complications and device failures. Operative data and angiographic and computed tomography images were collected to assess the complications and treatment strategy.

**Results:**

A total of 198 patients with 275 puncture sites underwent pEVAR with the 6-Fr Perclose Proglide. The procedure was successful in 178 patients (89.9%), whereas PRC occurred in 20 cases (10.1%), including 10 device failures and 10 vascular complications. An extra manual ancillary compression was conducted in 7 patients, one more device was used in 8 patients, and surgical repair of the femoral artery was performed in 5 patients. PRC had a tendency to occur in patients with body mass index (BMI)>30 kg/m^2^ (p = 0.021), thoracic stent grafts (p = 0.038), common femoral artery (CFA) calcification (p = 0.001), CFA depth>4 cm (p = 0.001), and sheath size>20Fr (p = 0.005). Device failure-related mortality was zero. None of the access sites had complications during the midterm follow-up.

**Conclusions:**

The pre-close technique with 6-Fr Perclose Proglide devices for pEVAR appears to be safe and effective with low technical failure and complication rates. Careful patient selection and proficiency in device manipulation might reduce the device related complications.

## Introduction

Endovascular aortic repair (EVAR) has been an option for the treatment of aortic diseases, such as dissections[1] and aneurysms[2,3]. Arteriotomy of the common femoral artery (CFA) is usually performed to deliver the stent grafts. Since the advent of “pre-close technique” advocated by Haas et al.[4], percutaneous EVAR (pEVAR) has been applied to decrease the morbidity of the groin incisions.

Six-Fr Perclose Proglide (Abbot vascular, Redwood City, USA) is one of these vascular suture devices that consists of one 3–0, unbraided, pre-tied slipknot. A greater than 90% success rate has been reported for this device, and the technique can save procedure time, reduce hospitalization duration, and promote early ambulation[5–7].

However because of inherent limitations and the learning curve involved in its use, several 6Fr Perclose Proglide-related complications (PRC) remain unsolved[8,9]. Accordingly, the current study was conducted to investigate the procedural success rate and predictors of PRC in pEVAR.

## Methods

### Patient data

The ethics committee of Fudan University approved this clinical observational study. It was a retrospective study and the patients' consent was not obtained. All the data were analyzed anonymously. A retrospective analysis was performed on patients undergoing EVAR in our center from December 2012 to November 2013. Patients were included if they were suitable for percutaneous interventions using the pre-close technique. Patients with previous groin surgery and inguinal arterial prosthesis in the access site, a severely tortuous iliac artery, anterior or posterior plaques (>50% circumference), calcified annulus and narrowed (diameter<6 mm) femoral arteries were excluded.

Medical data for demographics, diagnosis, treatment modalities, outcomes, and follow-ups were retrospectively analyzed. In all subjects, the depth of the puncture sites and CFA calcifications was measured by ultrasonography (US) or computed tomographic angiography (CTA) before the operation.

### Surgical procedures

All operators were familiar with the use of the Perclose Proglide devices and have undergone training. All operators were experienced and have handled 20 successful sutures using this device.

Patients were operated on under general anesthesia. Prior to puncturing the CFA, we used US or CT data to mark the sites free of ring-shape calcification between the bifurcation and the groin ligament. After a small incision in the skin at the site, we expanded the superficial fascia or fat tissue using a vascular clamp before puncture.

Heparin was intravenously administered at 1 mg/kg dose. Arterial puncture using the modified Seldinger technique (not through the posterior wall of the artery) was performed. Prior to deployment of the first Perclose Proglide device, we performed a femoral angiogram to reconfirm the vessel size, calcium deposits, and tortuosity.

The two devices were deployed in the standard manner according to the protocol of the pre-close technique[10,11], but we rotated the devices left and right from the entrance axis approximately 30° each (60° between them). Stent graft selection was based on the preferences of the surgeons and vascular anatomy.

Prior to the insertion of large-bore stent-graft delivery sheaths, we often perform a progressive pre-dilatation with 6Fr-10Fr sheaths. At the end of the pEVAR procedure, we carefully removed the entire sheath system and allowed the potential blood debris out. We checked the pedal pulses immediately after the closure and ensured that there were no changes compared to the preoperative findings. We administered protamine sulfate after checking for a healthy distal pulse.

### Outcome definitions

The primary success is defined as good device performance and femoral arterial hemostasis without any open repair conversion.

Bleeding is defined and classified according to the Bleeding Academic Research Consortium (BARC) criteria[12] at the access site after two device suturing pre-operatively or within 30 days afterwards. Vascular complications also included the access vessel dissection, arteriovenous fistula, and stenosis or stricture that leads to extremity ischemia or severely limited flow confirmed by imaging check.

Device failures involve failure to preload the sutures, suture rupture or tearing out, and insufficient tightening of the knots.

### Follow-up

Patients who underwent pEVAR were followed up at 30 days, 3 months and 6 months after discharge. During imaging follow-up, we not only screened the outcome of the aortic stent grafts but also evaluated the access to rule out late PRC events, such as stenosis and pseudoaneurysm.

### Statistical analysis

Demographics and background data from patients were descriptive. Continuous data were summarized as the mean ± standard deviation, and categorical data were described as counts and percentages. Differences between the success group and PRC group were analyzed using a combination of Pearson Chi- square test and Student’s t test for categorical and continuous data, respectively. Linear regression analysis was performed on the incidence of PRC. Multivariate analysis was performed to assess the impact of various variables on the PRC. Statistically significant difference was considered at P value<0.05. Statistical Package for Social Sciences for Windows (SPSS 19.0, Chicago, IL, USA) was used for data processing and statistical analyses.

## Results

### Patient characteristics

**Fig 1:**
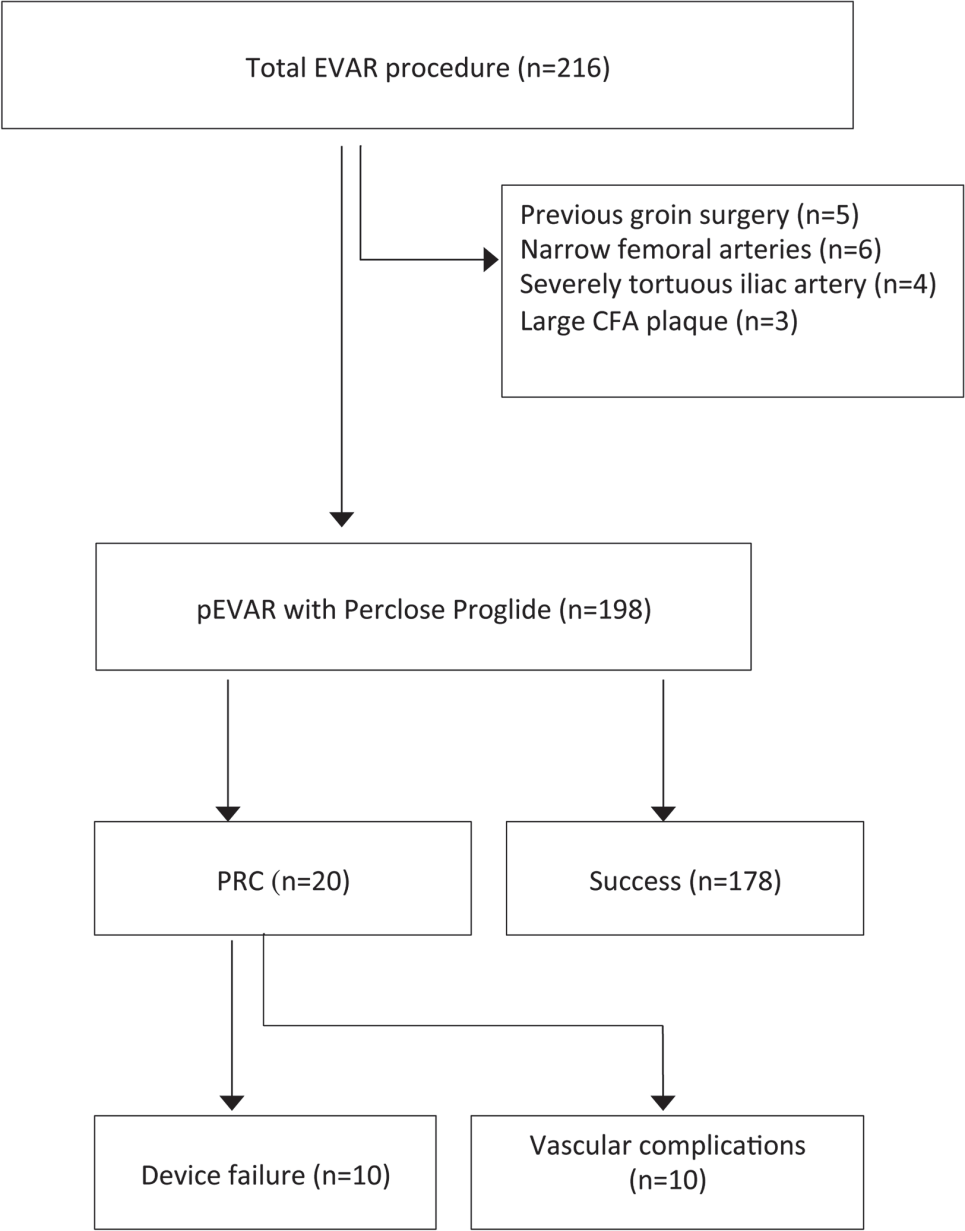
The Flowchart of this study. The flowchart depicts the inclusion and the exclusion of the study population in this study and the occurrence of Perclose Proglide-related complications.

We performed EVAR in 216 patients and deployed all stent grafts successfully during the study period, and 18 patients were ineligible for percutaneous access. We assessed 198 consecutive pEVAR patients (160 men and 38 women, mean age 62.2±13.0 years)(see Fig 1). Details of demographics and lesion data before the operation were displayed in Table 1.

**Table 1:** Demographics characteristic of the Patients.

Variables	N	%
Age (year)	62.2±13.0	
Gender		
Men	160	80
Women	38	20
BMI (kg/m^2^)	27.2±5.3	
Hypertension	142	71.7
DM	54	27.3
CAD	63	31.8
Smoking	96	48.5
Stent cover site		
Thoracic	121	61.1
Abdominal	77	38.9
Etiology		
AD	96	48.5
AA	90	45.5
IA	4	2
Endoleak^a^	8	4
CFA depth^b^ (cm)	3.2±2.0	
CFA calcification^b^	70	35.4

Continuous data are presented as the mean±standard deviation; categorical data are given as counts (percentage). DM:Diabetes mellitus; CAD: coronary artery disease; AD: Aortic dissection; AA: Aortic aneurysm; IA: Iliac aneurysm; CFA:common femoral artery.

a:Endoleak following previously thoracic EVAR

b: Data from the CTA image measurement of the patients before the operation.

### PRC and treatments

**Fig 2:**
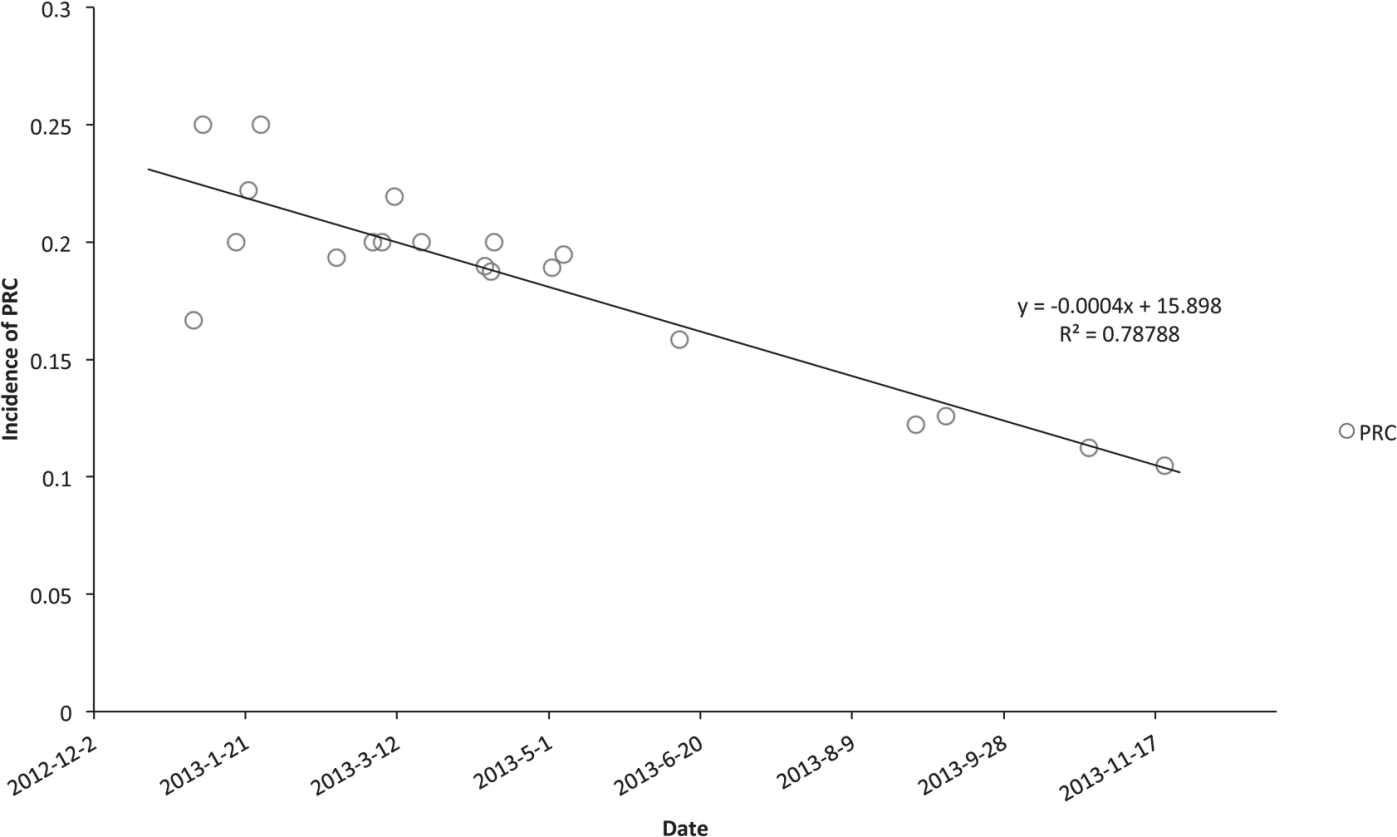
The PRC incidence and date. Simple linear regression analysis is performed to demonstrate the decline tendency of PRC incidence.

Total pEVAR was successful in 178/198 cases (89.9%) and in 255/275 access sites (92.7%). However, 10 vascular complications and 10 device failures occurred in pEVAR patients (10.1%). With the accumulation of manipulating experience, the incidence of PRC linearly decreased (y = -0.0004x+15.898, R^2^ = 0.78788)(Fig 2).

**Fig 3:**
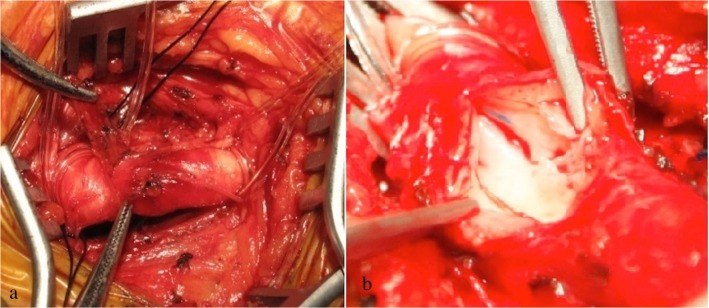
Open surgery at the access site. This series of photographs illustrates the twist shape of CFA when we converted to open surgery. The anterior and posterior artery walls were sutured together, and the CFA was occluded (a). The suture knot was located in the lumina (b).

Bleeding was addressed by manual compression in 4 cases, 3 access sites with vessel stricture or dissection finally induced cut-down conversion, and 7 device failures required another Perclose Proglide (see Table 2). One access artery dissection was indicated by radiography after suturing the access site, and the posterior and anterior arterial walls were sutured together after conversion (see Fig 3). We fixed the intima using the 7–0 Prolene thread and repaired the puncture orifice without a patch.

**Table 2:** Perclose Proglide related complications and treatments during pEVAR.

Complications (n)	Treatments (n)		
	Compression	Cut-down	Another device
Vascular complications (10)	4	5	1
Bleeding (6)	4	1	1
Minor bleeding (5)	4		1
Major bleeding (1)		1	
Dissection (1)		1	
Stenosis or stricture (3)		3	
Device failures (10)	3	0	7
Failure to preload sutures (3)			3
Suture rupture or tearing out (2)	1		1
Insufficient to tighten knots (5)	2		3

### PRC and factors

Between the PRC group and the success group, etiology of the lesions, and baseline demographics including age, gender, hypertension, diabetes mellitus, coronary arterial disease, and smoking were similar (P>0.05). However, BMI, stent cover site, CFA calcification, CFA depth, and sheath size showed significant differences between the two groups (P<0.05)(see Table 3).

**Table 3:** Factors associated with PRC.

Variables	PRC (n = 20)	Success (N = 178)	*P* value
Age (year) †	65.5±10.2	61.8±13.3	0.2406
Gender (man)	13(65%)	147(82.6%)	0.1110
BMI (kg/m^2^) †	29.5±5.5	27.0±5.2	0.0410
Hypertension	15(75%)	127(71.3%)	0.7310
DM	6(30%)	48(27.0%)	0.7727
CAD	3(15%)	60(33.7%)	0.0885
Smoking	13(65%)	83(46.6%)	0.1191
Stent cover site (Thoracic)	13(65%)	64(36.0%)	0.0115
Etiology (AD)	7(35%)	89(50%)	0.2031
CFA depth (cm) †	4.1±2.3	3.0±1.9	0.0227
CFA calcification	14(70%)	56(31.4%)	0.0006
Sheath size (>20Fr)	6 (40%)	17(7.9%)	0.0001

† Indicates continuous data were analyzed using the Student's t test, other categorical data using the Pearson Chi-square test.

Further, compared with the other group, the PRC incidence was higher in patients with BMI>30 kg/m^2^ (P = 0.011), CFA calcification (p = 0.003), thoracic stent grafts (P = 0.021), CFA depth>4 cm (P = 0.038), and sheath size>20Fr (P = 0.019). Multiple logistic regression analysis identified BMI>30 kg/m^2^ (P = 0.021), CFA calcification (P = 0.003), thoracic stent grafts (P = 0.038), CFA depth>4 cm (P = 0.001), and sheath size>20Fr (P = 0.005) as independent predictors of PRC (see Table 4).

**Table 4:** Logistic regression analysis of preoperative classified variables for PRC.

Variables			Univariate	Multivariate		
			*P* value	*P* value	OR	95% CI
BMI	>30kg/m^2^	11/60	0.011	0.021	4.020	1.228–13.156
	≤30kg/m^2^	9/138				
CFA depth	>4cm	9/47	0.038	0.001	8.138	2.353–28.148
	≤4cm	11/151				
Stent cover site	Thoracic	13/77	0.021	0.038	4.429	1.086–18.065
	Abdominal	7/121				
Sheath size	>20Fr	6/23	0.019	0.005	6.318	1.731–23.054
	≤20Fr	14/175				
CFA calcification	<50%*	13/70	0.003	0.001	6.790	2.132–21.620
	Free	7/128				

* indicates circumferential portion of calcium plaques at the access sites.

### Late events

During the follow-up, postoperative CTA or US was performed in all patients. One patient died of heart failure in the 3^rd^ month, which was unrelated to the PRC. None of the access sites had stenosis or stricture or other groin complications at 3 and 6 months.

## Discussion

Perclose Proglide is the second-generation suture-based vascular closure device that has been used for a decade. We can easily obtain the benefit of reducing hospital stay and improving patients’ early ambulation[11,13]. Further, Proglide has the special superiority of suturing large caliber orifices in vessels, especially when the “pre-close technique” is applied during pEVAR procedures, which usually require a large sheath (range 12Fr to 26Fr profiles) to ensure transporting and deploying the aortic stent graft. The pre-close technique is efficient for achieving hemostasis in the femoral artery[14]. The immediate high success rate (89.9%) in our center is comparable to those reports [8,9,11,14–28]. However, PRC or other events were also reported in 10% to 20% of these patients in the literature[29,30], and 10.1% at our center (Table 5).

**Table 5:** PRC and factors referred in recent studies of Perclose Proglide in pEVAR.

Study	Success	Pts./PRC	BMI	Sheath	Exp.	Dia.	Cal.	Dep.
Zakko[17] 2014	92.0%	355/29	-	+		+		+
Ye[18] 2014	96.5%	113/4		+				
Nelson[16] 2014	94.0%	50/3			+			
Kim[14] 2013	93.2%	367/25						
Jabori[8] 2013	92.1%	76/6						
Bechara[9] 2013	81.8%	99/18	-	-	+	-	-	
Bensley[19] 2012	95.8%	168/7				+		
Al-Khatib[20] 2012	95.8%	24/1				+		
Wei[21] 2012	93.2%	103/7						
Ni[22] 2011	90.6%	85/8			+			
Krajcer[23] 2011	97.4%	38/1						
Zhang[24] 2010	91.2%	68/6						
Grenon[25] 2009	86.7%	15/2						
Smith[26] 2009	90.9%	11/1	-				-	
Lee[27] 2008	94.5%	292/16						
Lee[15] 2007	94.3%	262/15	+		+		+	+
Dosluoglu[28] 2007	82.3%	17/3						

“+” Indicates the relationship between factors and PRC is positive, and “-”indicates negative.

Pts. (Patients), Exp. (Experience), Dep. (Depth), Dia. (Diameter), Cal. (Calcification).

Bleeding is a common vascular complication (6/10) during pEVAR; however, it is not life-threatening and can be successfully treated with manual compression (4/6) leading to successful hemostasis[8,29]. Compared with patients in percutaneous coronary intervention, those in this series were administered an antagonist to heparin at the end of the procedure, which may be one reason that they were free of anticoagulation postoperatively [8,29,31]. Conversion mostly occurred for stricture or stenosis complications, and the incidence in this series is lower than that in the previous report (5/198 vs. 4/78)[8].

We cannot ignore the value of the learning curve during pEVAR, which is likely related to the device failures[9,16,22]. The first multicenter randomized controlled trial indicated that training and experience with the pre-close technique were important for ensuring successful outcomes[16]. In our center, device failure occurred mostly in the early days (see Fig 2). Therefore, we have regular training for vascular surgeons to improve their familiarity with properly deploying the devices, and they have handled 20 cases successfully. However, another larger pEVAR study has produced a different result regarding the relationship between failure and experience[32]. Surgeons with more experience are prone to risking the less favorable anatomy and may have a high failure rate[5].

Obesity represents a risk factor that is significantly associated with the incidence of complications during pEVAR[33,34], other than in conventional surgical access to the femoral site. We found that the incidence of PRC in the BMI>30 kg/m^2^ group was relatively high (18.3%). Therefore, Jean-Baptiste et al. [6] chose patients with moderate obesity (BMI <35 kg/m^2^) for pEVAR. However, several negative results in the literature revealed that BMI was not related to PRC[9,17,26]. In fact, the general state of weight or BMI was not the main reason, but rather, the depth of the subcutaneous tissue in the groin was important[15,17], which is another risk factor for PRC revealed in our study. Zakko et al. [17] reported that the CFA depth was significantly greater in obese patients (50±20 mm *vs*. 30±13 mm; P <. 0001). If patients have thick fat tissue at the puncture sites (depth of CFA>4 cm), pushing down of the slipknot is usually difficult and may result in loosening of the knots.

Numerous previous studies have mentioned that the success rate is significantly related to the caliber of the sheath[5,11,17,18,29]. Lee et al. [15] concluded that large sheaths had low technical success rates among the subset of sheath sizes. Kim et al. [14] also found 7 of 8 closure procedure failures in cases involving sheaths over 16Fr and believed that procedural failure might be related to large sheath sizes. To date, we consider the sheath size>20Fr as a predictor of PRC in pEVAR, and it is reasonable to infer that PRC occurs more often in thoracic stent grafts, which usually use delivery sheaths larger than 18Fr[17,18]. In the reviewed articles[17,19,20], the CFA diameter was considered another predictor of PRC, which implied that sheath size and vessel diameter might co-contribute to the result.

In one meta-analysis, the quality of the artery was found to be a greater predictor of failure than the sheath size itself[5]. The use of the Proglide device is not considered when the CFA has a large plaque (>50% circumference) or ring-shape calcification. Manunga et al. [35] found that cases of patients with >50% anterior wall calcification had a higher failure rate than calcification-free patients. We also found that even the patients with <50% CFA calcification tend to develop PRC, compared with patients who are free of CFA calcification. In the case of severe calcification, we would most likely perform a CFA endarterectomy instead.

During midterm follow-up, late PRC such as infection, thrombosis, stricture, or occlusion are rare[15]. One of our patients died of heart failure at 3 months in this study. The mortality of the pEVAR procedure was low (<0.2%) and was usually related to the underlying comorbidities of the patients but not the devices themselves[28,34,36]. However, clinical long-term follow-up is imperative for pEVAR patients.

## Conclusions

The pre-close technique with two 6F Perclose Proglide devices for pEVAR appears to be safe and effective. Obesity (BMI>30 kg/m^2^), thoracic stent grafts, deep CFA (>4 cm), CFA calcification, and large sheath size (>20Fr) predict the risk for technical failure and vascular complications. Careful patient selection and proficiency in device manipulation might reduce the device related complications.

## Limitations

This study is limited by its retrospective nature. We did not compare the efficacy between Perclose Proglide and other devices. Furthermore, different criteria for PRC may alter the incidence of the adverse event and thereby impair the results.
